# Nano-Structuration of WO_3_ Nanoleaves by Localized Hydrolysis of an Organometallic Zn Precursor: Application to Photocatalytic NO_2_ Abatement

**DOI:** 10.3390/nano12244360

**Published:** 2022-12-07

**Authors:** Kevin Castello Lux, Katia Fajerwerg, Julie Hot, Erick Ringot, Alexandra Bertron, Vincent Collière, Myrtil L. Kahn, Stéphane Loridant, Yannick Coppel, Pierre Fau

**Affiliations:** 1 LMDC, INSA/UPS Génie Civil, 135 Avenue de Rangueil, CEDEX 4, 31077 Toulouse, France; 2 LCC-CNRS, UPR8241, 205 Route de Narbonne, CEDEX 4, 31077 Toulouse, France; 3 Université de Toulouse, UT3 Paul Sabatier, 118 Route de Narbonne, CEDEX 04, 31062 Toulouse, France; 4 LRVision SAS, 13 Rue du Développement, 31320 Castanet-Tolosan, France; 5 Univ. Lyon, Université Claude Bernard-Lyon 1, CNRS, IRCELYON-UMR 5256, 2 av. A. Einstein, 69626 Villeurbanne, France; 6 LPCNO-INSA, UMR5215, 135 Avenue de Rangueil, CEDEX 4, 31077 Toulouse, France

**Keywords:** WO_3_ nanoleaves, hetero nanomaterials, localized hydrolysis, photocatalysis, NO_2_ abatement

## Abstract

WO_3_ is a known photocatalytic metal oxide frequently studied for its depollution properties. However, it suffers from a high recombination rate of the photogenerated electron/holes pair that is detrimental to its performance. In this paper, we present a new chemical method to decorate WO_3_ nanoleaves (NLs) with a complementary metal oxide (ZnWO_4_) in order to improve the photocatalytic performance of the composite material for the abatement of 400 ppb NO_2_ under mild UV exposure. Our strategy was to synthesize WO_3_·2H_2_O nanoleaves, then, to expose them, in water-free organic solution, to an organometallic precursor of Zn(Cy)_2_. A structural water molecule from WO_3_·2H_2_O spontaneously decomposes Zn(Cy)_2_ and induces the formation of the ZnO@WO_3_·H_2_O nanocomposite. The material was characterized by electronic microscopy (SEM, TEM), TGA, XRD, Raman and solid NMR spectroscopies. A simple thermal treatment under air at 500 °C affords the ZnWO_4_@WO_3_ nanocomposite. The resulting material, additionally decorated with 1% wt. Au, presents a remarkable increase (+166%) in the photocatalytic abatement of NO_2_ under UV compared to the pristine WO_3_ NLs. This synthesis method paves the way to the versatile preparation of a wide range of MOx@WO_3_ nanocomposites (MOx = metal oxide).

## 1. Introduction

The development of new methods for the preparation of complex nanocomposite materials (MOx@WO_3_, MOx = metal oxide) is a prerequisite for increasing the performances of materials dedicated to catalytic, photocatalytic or gas sensor applications [1,2,3]. The building of complementary heterostructures is useful for improving their physical or chemical properties such as band gap modulation, facile generation of electron–hole pairs, low recombination rate, longer carrier’s lifetime, or larger absorption of the electromagnetic spectrum [4]. These properties are, moreover, generally enhanced for nanostructures, having a strong interfacial contact with their supporting material. The quality of material interface plays a major role in the diminution of charge carriers’ recombination due to surface defects [5,6]. It is, therefore, of prime importance to develop chemical methods allowing for the building of heterostructures presenting an optimum interface between each of the components. Semiconducting oxide powders exposed to UV-vis wavelengths, or to the solar spectrum, are promising photocatalytic materials for air pollution reduction [7]. The operating principle is based on their ability to generate electron/holes pairs that can induce oxidizing species, leading to pollutant abatement by oxidation or mineralization mechanisms [8]. The combination, in a close chemical interaction, of a photocatalytically active metal oxide such as WO_3_ [9] with another photocatalytic material based on zinc oxide [10], is expected to improve the photocatalytic performance of the nanocomposite [11]. WO_3_ is indeed a common photocatalyst [12] that is extensively investigated for gas sensors [13,14], oxidation of water [15], and efficient adsorbent for pollutant removal from water [16].

However, the preparation of ZnO nanoparticles (NPs) in close contact with WO_3_ supports is not straightforward. For example, the controlled hydrolysis of Zn(Cy)_2_ in an organic solvent with 2 molar equivalent (equiv.) of water is a method developed in our team to prepare controlled colloidal ZnO NPs [17,18]. Zn(Cy)_2_ strongly reacts with water molecules in organic solvents to spontaneously afford ZnO NPs. Additionally, this reaction is exothermic enough to yield well-crystallized wurtzite nanostructures at room temperature. In this method, colloidal NPs are stabilized by the addition of molecular ligands such as long chain alkylamines, carboxylic acid or a combination of the two. However, these ligands may become unwanted surface pollutants and detrimental to the useful properties of ZnO nanomaterial, as in the case of applications that rely on surface chemical reactions [19]. These ligands only can be removed by these washing and centrifugation methods, but strongly bound ones either persist or are transformed into undesired carbon species remaining on the ZnO surface.

In this work, we have developed a new chemical implementation to prepare nanostructured ZnO supported on WO_3_ nanoleaves (NLs) without the help of any additional ligands. This method allows the formation of intimate interfaces between the oxides that are beneficial to the physicochemical properties of the metal oxide composite. In this new method, we have taken profit of the structural water molecules present within WO_3_·2H_2_O NLs to allow their very localized reaction with an organic solution of the Zn(Cy)_2_ precursor. We have achieved here the controlled growth of ZnO nanostructures at the surface of the NLs. The WO_3_ NLs play three complementary functions: (i) they present structural water molecules that in situ react with Zn(Cy)_2_, (ii) they stabilize the ZnO nanocrystals, and (iii) they present a leaf shape that is suitable for catalytic or photocatalytic reactions. The ZnO@WO_3_ powders are further transformed into ZnWO_4_@WO_3_ by a simple thermal treatment at 500 °C in ambient air. The WO_3_ NLs, and those prepared with ZnWO_4_ nanostructures, are eventually decorated with Au NPs (1% wt.) by a simple photodeposition method in solution. The hetero nanomaterials of this study were tested as photocatalysts for the degradation of 400 ppb NO_2_ under UV. Due to the very close interface between ZnWO_4_ and the WO_3_ support, the complex Au-ZnWO_4_@WO_3_ composite of this study exhibits a great improvement in NO_2_ abatement (+166%) compared to bare WO_3_ NLs.

## 2. Materials and Methods

### 2.1. Materials

Sodium tungstate dihydrate (Na_2_WO_4_, 2H_2_O) was purchased from Sigma Aldrich and used without further purification. Dicyclohexyl zinc (Zn(Cy)_2_) was purchased from Nanomeps SA and stored in argon atmosphere in a glove box at −35 °C before use. Toluene was purchased from Sigma Aldrich and dried at least 24 h over activated molecular sieve in a glove box prior to use. Acidic exchange resins (DOWEX-50WX2, 100–200 mesh) were purchased from Sigma Aldrich. Chloroauric acid (HAuCl_4_·2H_2_O) for gold decoration was purchased from Sigma Aldrich and used without further purification.

### 2.2. Preparation of WO_3_·xH_2_O NLs (x = 2, 1, 0)

The preparation of NLs of tungstic acid dihydrate (WO_3_·2H_2_O, monoclinic) is derived from the method described by Chemseddine [20] and modified by Choi [21]. The synthesis protocol is divided into two steps: (i) a solution of Na_2_WO_4_, 2H_2_O (0.4 M, 660 mg in 5 mL distilled H_2_O) was allowed to flow at constant rate through the acidified ion exchange resin placed in a glass column. Five successive additions of 10 mL H_2_O in the column were necessary to collect the eluent at pH < 2 in a beaker and obtain approximately 30 mL of a limpid metatungstic acid solution (H_2_WO_4_); (ii) the solution was placed in a sealed vial and placed in an orbital shaker at 150 rpm (Benchmark Scientific BT302-E) at room temperature for 72 h to allow the ageing process and crystallization of WO_3_·2H_2_O NLs (yellow solution). Due to NLs presence, a rheoscopic fluid was obtained. Amorphous and water-soluble fractions (tungstic acids) of the solution were removed by three successive washing and centrifugation steps of the yellow solution with distilled water. A final washing with pure ethanol facilitated the drying of the resulting powder under a moderate primary vacuum. The NLs’ mean size and standard deviation were estimated by statistical analysis (ImageJ software, NIH, MD, USA) of SEM images of WO_3_·xH_2_O powder deposited on a conductive silicon support. The H_2_O content of WO_3_·xH_2_O NL can be decreased from 2 to 1 and 0 by a simple thermal treatment under ambient air (at 100 and 350 °C respectively).

### 2.3. Decoration of WO_3_·2H_2_O NLs by ZnO NPs

WO_3_·2H_2_O (300 mg) powder was finely ground with an agate mortar and entered in a glovebox. The powder was then dispersed in 20 mL of dry toluene inside a damp-proof glass container and sonicated for 20 min. Once the powder was well dispersed, a yellow rheoscopic solution (solution A) was obtained. In the glove box, solution B was prepared by adding Zn(Cy)_2_ (300 mg) in 5 mL of dried toluene. Solution B was gently added to solution A under continuous stirring. Upon addition, the solution turned into green due to the chemical reduction of W^6+^ to W^5+^ ions induced by the highly oxophilic zinc precursor [22]. After 3 h of stirring, the green precipitate was collected by centrifugation, washed three times with dry toluene (glovebox) and finally with pure ethanol before drying under primary vacuum.

### 2.4. AuNPs Decoration of WO_3_ and ZnWO_4_@WO_3_·H_2_O NLs

The powders were decorated with AuNPs obtained by photodeposition of chloroauric acid precursor (HAuCl_4_·3H_2_O) exposed to UV lamp (Xenon lamp, 100 W, 17 W/m^2^). Typically, 200 mg of powder was dispersed in 200 mL of ultra-pure water and sonicated for 20 min. Then, 4 mg of gold precursor (1% wt. Au) was added to the solution and sonicated for a further 2 min at the exclusion of light. The homogeneous solution was then placed under the UV source under stirring for one hour. The green suspension turned grey upon Au deposition. Powders were centrifuged and washed three times with deionized water and dried 1 h under primary vacuum at room temperature.

### 2.5. Characterization

Transmission electron microscopy (TEM) images were obtained with a JEOL 1400 transmission electron microscope operating at 120 kV. A JEOL JEM-ARM 200f (JEOL Ltd., Tokyo, Japan) operating at 200 kV was employed to collect high-resolution TEM images. The system is equipped with a probe corrector and a STEM HAADF detector (scanning TEM high angle annular dark field) for Z-contrast and also possesses an EDX (energy-dispersion X-ray) analysis system. Field emission scanning electron microscopy (FESEM) images were obtained using a JEOL JSM-6700F microscope operating at 10 kV. Solid-state nuclear magnetic resonance spectroscopy (NMR) experiments were recorded on Bruker Avance 400 III HD (Bruker Corp. Billerica, MA, USA), spectrometer operating at magnetic fields of 9.4 T at room temperature. Samples were packed into 1.3 mm zirconia rotors and were spun at 50 kHz. ^1^H MAS were performed with the DEPTH pulse sequence and a recycle delay of 3 s. ^1^H MAS with rotor synchronous dipolar filtering and spin echo excitation (DF-SE) were acquired with a filtering time of 9.6 ms. The 1D and 2D ^1^H-^1^H double-quantum (DQ) MAS experiments were recorded with a back-to-back (BABA) recoupling applied for 2 rotor periods. ^1^H liquid-state NMR spectra were recorded with a Bruker Avance 400 III spectrometer (9.4 T) at 298K in deuterated toluene. Chemical shifts were referenced to TMS. Powder-diffraction patterns were obtained using SEIFERT XRD 3000 TT X-Ray Diffractometer (Seifert X-Ray, Germany), with Cu-Kα radiation, fitted with a diffracted-beam graphite monochromator. The data were collected in the 2θ configuration between a 10 and 70° angle. Thermogravimetric analysis (TGA) was performed using a Setaram thermobalance (Setaram Engineering, France) with a ramp of 10 °C/min in the 30–500 °C range under ambient air. MicroRaman spectra were recorded with a LabRAM HR spectrometer (Horiba, Kyoto, Japan) using the exciting line at 514.53 nm of an Ar ^+^ ion laser, 50 times magnification objective and CCD open electrode detector cooled down to −75 °C. The spectral resolution was 4 cm^−1^. The laser heating was negligible with the power used (100 µW). In situ studies were performed in a THMS600 cell coupled with a TMS94 programmer (Linkam Scientific, Salfords, UK). The temperature gradient between the heating sole and in the upper part of the powder sample was previously determined and corrected.

### 2.6. Photocatalytic Activity

The photocatalytic powders (20 mg) were dispersed in ethanol and spray-coated on borosilicate glass substrates (50 mm × 100 mm × 5 mm). The substrate was dried at ambient temperature up to full ethanol evaporation to obtain a uniform coating. The final dry content of photocatalyst at the surface was equal to 4.0 ± 0.5 g/m^2^ (20 mg on 50 cm^2^ glass sample). This value was found to be the optimal photocatalyst dry content according to a previous study [23]. Samples were aged for at least 24 h in the dark before performing the photocatalytic tests.

### 2.7. NO_2_ Degradation

The experimental setup, adapted from standard ISO 22197-1:2016, is described in more details in the reference [23]. The polluted air stream, with a NO_2_ concentration of 400 ppb, entered the reactor at a flow rate of 1.50 L/min. The desired relative humidity level (50%) of the polluted air stream was obtained by mixing the dry air flow with humidified air by passing through a gas washing bottle containing deionized water. The NO_2_ concentration was measured by a chemiluminescent analyzer (model AC32M, Envea (ex-Environment SA)). The experiments were carried out under UV light at 1 W/m^2^. The light intensity was measured using a radiometer (Gigahertz-Optik), and emission spectra were recorded with a miniaturized spectrophotometer (OceanView). UV light was obtained by using a blacklight blue fluorescent tube (NARVA Blacklight Blue T8 18 W-073). Its spectral irradiance distribution between 200 and 800 nm is given in Figure S1. The photocatalytic activity of the functionalized samples was assessed through the photooxidation of NO_2_. The NO_2_ degradation (%) was calculated according to Equation (1).

(1) $${\mathrm{NO}}_{2}\;\mathrm{degradation}\;\left(\%\right)=100\times \frac{{\left[{\mathrm{NO}}_{2}\right]}_{\mathrm{initial}}-{\left[{\mathrm{NO}}_{2}\right]}_{\mathrm{final}}}{{\left[{\mathrm{NO}}_{2}\right]}_{\mathrm{initial}}}$$

where [NO_2_]_initial_ is the concentration measured (ppb) by the analyzer at the exit of the reactor before light activation once the steady state was established; [NO_2_]_final_ is the concentration measured (ppb) at the exit of the reactor after light activation (averaged over the last 10 min).

## 3. Results and Discussion

### 3.1. Characterization of WO_3_·xH_2_O (x = 0, 1, 2)

**Figure 1:**
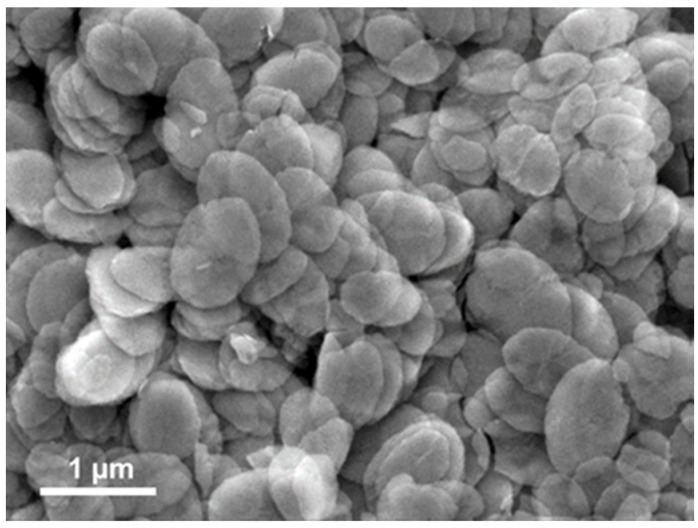
SEM image of pristine WO_3_·2H_2_O (**1**) NL.

The SEM image of WO_3_·2H_2_O NLs (compound 1 denoted (**1**)) resulting from the condensation of tungstic acid (see Material and Methods section for experimental details) is shown in Figure 1. The powder contains leaf-shaped particles with the dimensions of 700 ± 200 nm long, 500 ± 200 nm large and approximately 30 ± 10 nm thick (according to measurements by SEM analysis of the particles). As shown by Choi et al. [21], WO_3_·2H_2_O NLs tend to grow with the (010) planes parallel to each other so that it generates two dimensional (2D) plate-like crystallites. The overlapping of several of the particles observed in the SEM images reveals the underneath ones by transparency, confirming, thus, the very low thickness of the leaves.

**Figure 2:**
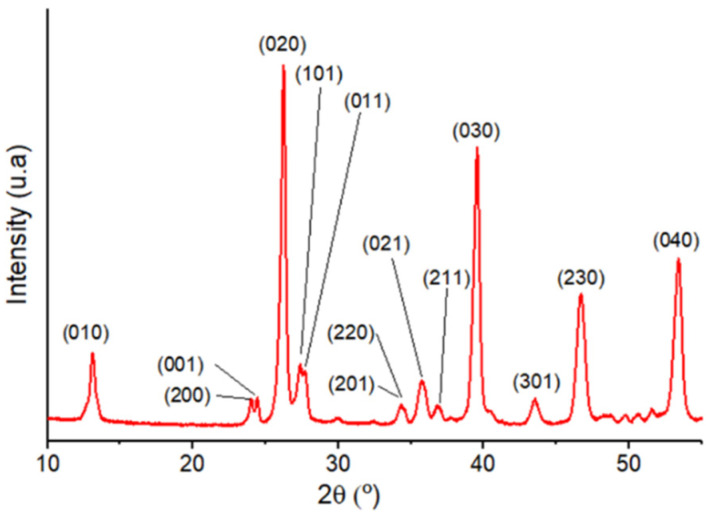
XRD diffractogram of (**1**) (WO_3_·2H_2_O) corresponding to the monoclinic structure (JCPDS 018-1420).

This agglomeration process driven by (010) planes is confirmed by the XRD pattern of the yellow powder, which corresponds to the monoclinic P2/m structure of WO_3_·2H_2_O (JCPDS card n°018-1420, Figure 2). A clear exaltation of the (020), (030) and (040) planes is characteristic of the crystalline structuration of the NLs.

Some authors working on the protonic conduction of (**1**) have compared the crystallographic structure of the dihydrate phase of WO_3_ to the monoclinic P21/n one of MoO_3_·2H_2_O [24,25]. The structure is lamellar and is described by single sheets made of [WO_5_-H_2_O] octahedrons in corner-sharing mode [26]. There are two different types of molecular water in the structure. W^6+^ cations are coordinated to five O^2−^ atoms and are located in the center of a square based pyramid formed by four equatorial oxygen atoms and one oxygen axially placed at the top (Figure S2). A water molecule is located in the axial position of the square base pyramid and corresponds to one of the tops of the octahedron [WO_5_-H_2_O]. This first type of water molecule is described as a “coordination” molecule in interaction with the oxygen atoms of the adjacent octahedron through hydrogen bonding. The other type of water molecules, or “interlamellar” water molecules, are located on the (020) plane and are in interaction with the top oxygen of [WO_5_-H_2_O] octahedron and the “coordination” H_2_O molecule of the previous octahedron. The (020) plane is, therefore, exclusively formed by interlamellar H_2_O molecules (Figure S2b). The larger crystallographic lattice employed to describe the dihydrate compound (**1**) (*a* = 10.57 Å, *b* = 14.12 Å and *c* = 10.67 Å with β = 90.5°) is a useful model to show the two different structural water molecules in the compound.

**Figure 3:**
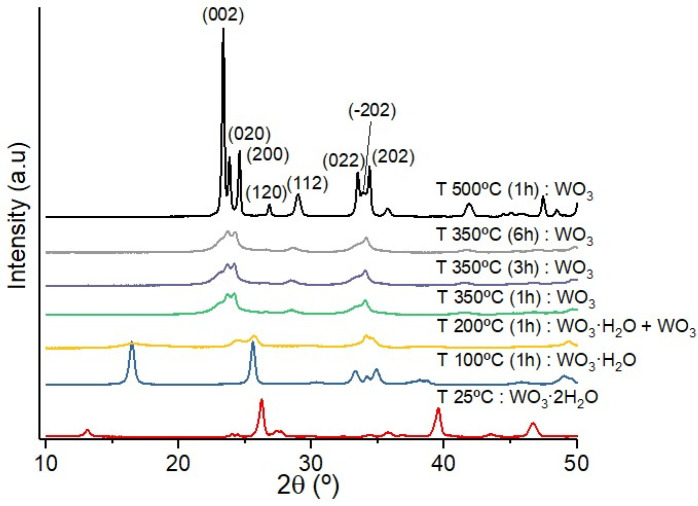
XRD analyses of (**1**) thermally treated up to 500 °C under air. The phase transformation shows the transformation of (**1**) to (**2**) at 100 °C and to (**3**) at 350 °C.

We have performed time-resolved XRD analyses of (**1**) placed on an integrated heating plate operated from 25 to 350 °C in ambient air (Figure 3). At 100 °C, the monohydrate phase WO_3_·H_2_O (compound **2**, denoted (**2**)) is formed, and above 200 °C the anhydrous WO_3_ compound (compound **3**, denoted (**3**)) begins to appear by a topochemical transformation [27,28]. The compound (**2**) corresponds to an orthorhombic structure (Pmnb, *a* = 5.2 Å, *b* = 10.7 Å and *c* = 5.1 Å, X-ray diffraction card number JCPDS No.43-0679) and is also constituted of a [WO_5_-H_2_O] octahedron [29]. Similarly to (**1**), the W atom is located in the center of the octahedron formed by five O atoms placed in a square base and one at the top of the pyramid, whereas one H_2_O molecule forms the opposite top of the octahedron. In that case, there are no inter-lamellar H_2_O molecules. The product (**3**) presents a monoclinic form (P21/n, *a* = 7.29 Å, *b* = 7.54 Å and *c* = 7.69 Å with β = 90.91°) of pure WO_3_. It is worth noting that the crystallinity of (**3**) is rather fair at 350 °C (mean size 17 ± 8 nm) and can be improved by a thermal treatment at 500 °C. At this temperature, the crystallite mean size increases up to 35 ± 15 nm (Figure 3), which is favorable to a better photocatalytic efficiency [15]. In addition, we observe a strong crystalline orientation along the *c* axis with an intense peak for (002) planes that reflects the anisotropic shape (leaf-like) of WO_3_ grains.

**Figure 4:**
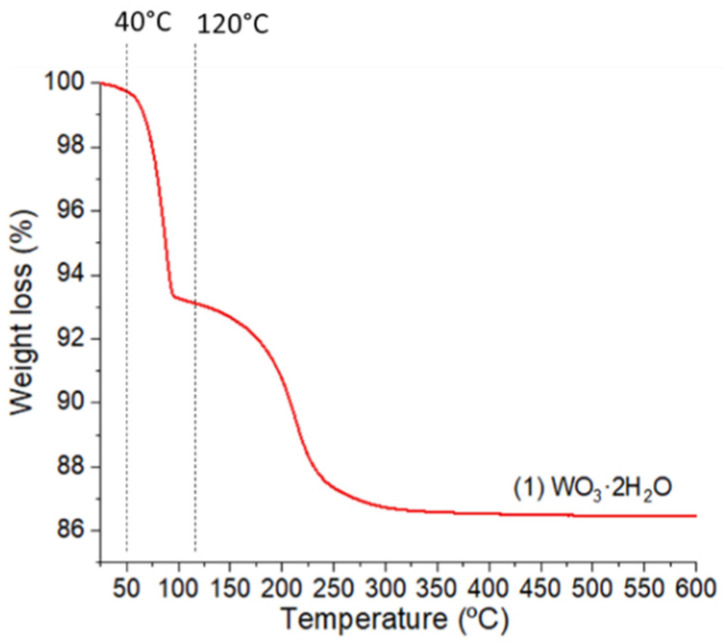
TGA analysis of (**1**) up to 500°C under air. Structural water molecule losses, respectively, appear at 40 and 120 °C with very close mean weight drops (−6.7 %).

By thermogravimetric analyses of (**1**), we have evidenced several characteristics of the dehydration steps (Figure 4).

The first dehydration step with a weight loss of 6.7% of the total weight begins at 40 °C and corresponds to the removal of the most labile water molecules in the structure of (**1**). After this first weight loss, a second one is observed beginning at 120 °C (−6.6%), and finally, the structure stabilizes at 320 °C. Up to 500 °C, no major change in the sample weight is noticed. The amplitude of the two weight losses are very close. Each of them corresponds to the departure of one water molecule from the structure of (**1**). These structural water molecules can be classified into two types: type 1 is related to the low bonding energy interlamellar molecules located on the (020) planes and characterized by an easy removal at low temperature; type 2 corresponds to the coordinated water molecules, which necessitate a higher thermal energy to be removed from the structure. The successive dehydration steps of (**1**) lead to the formation of (**2**) and (**3**), as shown by XRD analyses.

**Figure 5:**
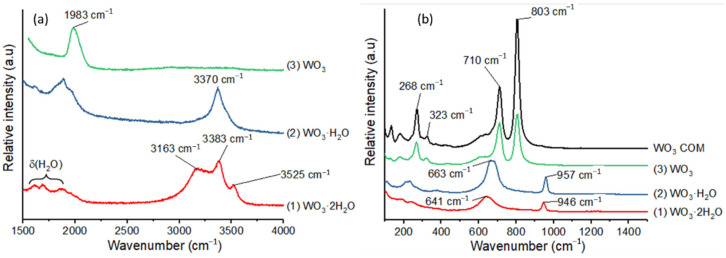
Raman spectra of (**1**), (**2**) and (**3**) (**a**) in the range 1500–4000 cm^−1^ and (**b**) in the range 100–1500 cm^−1^.

Raman analyses performed on (**1**), (**2**) and (**3**) allow characterization of the different water molecules in the structures (Figure 5) [4,30,31].

In order to study the nature of water molecules in (**1**) and (**2**), we have analyzed the wavenumber range between 1500 and 4000 cm^−1^ [32]. First, (**1**) exhibits a massif with three bands corresponding to ν (H_2_O) stretching vibrations at 3163 cm^−1^, 3383 cm^−1^ and 3525 cm^−1^ (Figure 5a), whereas a single band at 3370 cm^−1^ appears for (**2**). In addition, second order vibrations and δ (H_2_O) bending mode appear in the range 1500–2000 cm^−1^ for (**1**) and (**2**). We also confirmed the temperature transitions by in situ heating of (**1**) in the Raman cell. The compound (**1**) is stable up to 50°C and transforms in (**2**) at 100 °C (Figure S3). The compounds (**1**) and (**2**) present similar features in the range 600–1000 cm^−1^ with two rather wide bands located at ca. 650 and 950 cm^−1^. The band at 950 cm^−1^ is ascribed to the stretching vibrational mode of the terminal W=O bond of the octahedra [33]. The band at 650 cm^−1^ corresponds to the W-O-W bridging bonds in the equatorial plane of the W atom. The Raman analysis clearly evidences the specific vibrational signatures corresponding to the two types of water molecules within WO_3_·xH_2_O (x = 2, 1). The type 1 water molecules (interlamellar ones) present specific H_2_O stretching bands at 3163 and 13,525 cm^−1^. The compound (**3**) obtained by a thermal treatment at 500 °C under air is clearly different from the other structures with two intense bands at 710 and 800 cm^−1^. Its Raman spectrum corresponds well with one of a commercial crystalline nano-powder of WO_3_ (Figure 5b).

**Figure 6:**
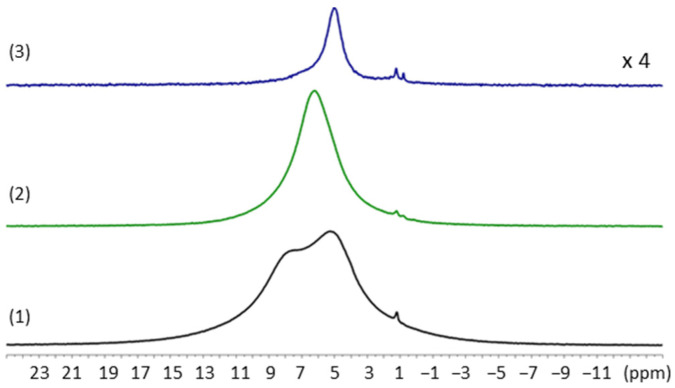
^1^H MAS NMR (ν_r_ = 50 kHz) spectra corresponding to compounds (**1**), (**2**) and (**3**). The intensity of (**3**) spectrum was increased 4 times for better comparison. The small sharp signal between 1 and 2 ppm corresponds to a residual pollution by ethanol solvent.

The compounds (**1**), (**2**) and (**3**) were also analyzed by ^1^H MAS (magic angle spinning) NMR spectroscopy at 50 kHz. This technique characterizes the hydrogen atoms present in the structure and their environment, and allows for differentiation in the WO_3_·xH_2_O compounds. Isolated hydroxyl groups with no interaction with other molecules will exhibit the smallest chemical shifts, i.e., around 1 to 2 ppm. Conversely, H_2_O molecules or hydroxyl groups involved in strong hydrogen bonding with neighboring molecules will present a shift close to 7 to 10 ppm. The ^1^H NMR spectra shows two close peaks at 7.7 and 5.2 ppm for (**1**) and a single peak at 6.2 ppm and 5.0 ppm for compounds (**2**) and (**3**), respectively (Figure 6). The contribution at *ca*. 5 ppm comes from water molecules and/or hydroxyl groups that are labile or engaged in weak hydrogen bonds (H-bond).

As expected, and compared to the other compositions, the H quantity is clearly the lowest in compound (**3**). However, (**1**) exhibits two peaks characteristic of the two water or hydroxyl groups in different chemical environments. In order to decipher the nature of these protons, a dipolar filtering and spin echo excitation (DF-SE) NMR experiment was used (Figure S4) [34]. Such an experiment reduces the resonance intensity of H atoms, having a strong dipolar coupling due to the proximity with other H atoms (as in a rigid H_2_O molecule). For (**1**), the overall ^1^H signal intensity has considerably decreased and a single large peak around 7.5 ppm remains. This indicates the presence of a majority of rigid structural water molecules in the sample. The residual proton signal at 7.5 ppm may correspond to some isolated hydroxyl groups engaged in a strong H-bond at the surface of the powder. For (**2**), a residual single peak appears at 6.5 ppm, and for (**3**), almost no signal is observed. Similarly to (**1**), the signal of rigid water molecules of the structural composition has vanished and only hydroxyl groups with moderate to strong H bonds remain. In (**3**), the quasi absence of signal accounts for the very little amount of H present in this sample after thermal treatment.

**Figure 7:**
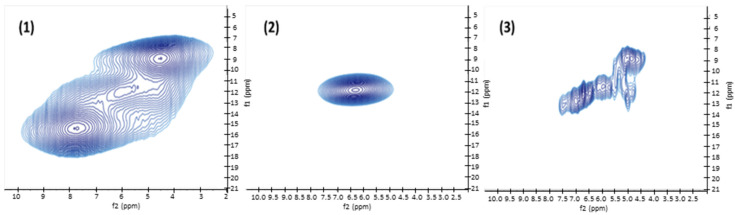
^1^H DQ MAS NMR (ν_r_ = 50 kHz) correlation spectra corresponding to compounds (**1**), (**2**) and (**3**).

The double quantum (DQ) MAS NMR experiment, which highlights the H atoms in dipolar interaction, was also measured for (**1**), (**2**) and (**3**) (Figure 7). The 1D version of this experiment helps to identify signals of H atoms that are spatially close, such as H_2_O or hydroxyl groups, that can be involved in hydrogen bonding interactions (Figure S5). Three correlations corresponding to different H atoms in close vicinity relationship can be clearly observed for (**1**). The first correlation centered at 8/16 ppm may correspond to rigid structural water molecules involved in the coordination in the [WO_5_-H_2_O] octahedron. The second strong correlation at 4.5/9.0 ppm is due to rigid H atoms and may correspond to interlamellar water molecules, which appear to be the most labile structural water of the structure (with the weakest H bond). Finally, the third and weak signal appearing at 6/12 ppm may correspond to few surface hydroxyl groups of WO_3_ or adsorbed water molecules from ambient air. For (**2**), a single and strong correlation signal at 6.2/12.4 ppm is attributed to the sole remaining structural water molecule in the [WO_5_-H_2_O] octahedron. For (**3**), which is prepared by thermal treatment at 500 °C, only weak interactions between H atoms are evidenced. They are attributed to some few physisorbed water molecules or surface hydroxyl groups due to the ambient air exposure of the WO_3_ NLs. This MAS NMR analysis helps to clearly differentiate the H atoms pertaining to water molecules engaged in the structuration of hydrated WO_3_ compounds (**1**) and (**2**). This technique correlates the Raman analysis of (**1**) and (**2**) and confirms the different spectroscopic signature of type 1 and type 2 water molecules within the structure. The type 1 water molecules are characterized by NMR responses always accounting for the presence of a higher density of neighboring water molecules. In sample (**2**), only type 2 water molecules remain in the structure that are characterized by a lower proximity with other water molecules.

### 3.2. Synthesis of ZnO@WO_3_ Nanocomposite by Reaction of WO_3_·2H_2_O with Dicyclohexylzinc Solution (Zn(Cy)_2_)

**Figure 8:**
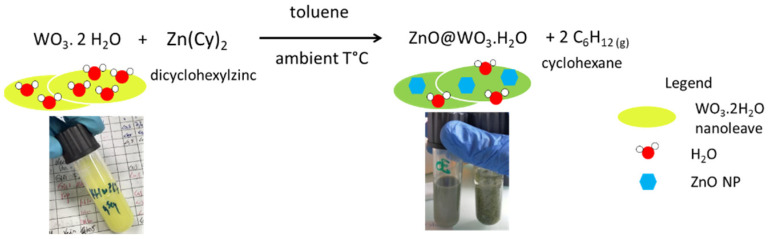
Reaction scheme for the preparation of the nanocomposite ZnO@WO_3_·H_2_O.

After the addition of 0.5 molar equiv. of Zn(Cy)_2_ to the yellow suspension of WO_3_·2H_2_O in toluene, it quickly turns green, indicating the reaction of the zinc precursor with the NLs. The color change is ascribed to the formation of W^n+^ (*n* < 6) species associated with the presence of oxygen vacancies [35,36]. This suggests that the oxophilicity of Zn^2+^ ions of the precursor is strong enough to remove oxygen atoms from the WO_3_ lattice and yield the first surface germs of ZnO. However, we observed by TEM that compounds (**2**) or (**3**), which present the lowest amounts of structural water, remain almost unaffected after their mixing with 0.5 molar equiv. of Zn(Cy)_2_. No visible ZnO nanostructures appear on the WO_3_ supports (Figure S6). This is confirmed by ^1^H NMR study, which reveals almost no consumption of the Zn(Cy)_2_ precursor even after a few hours of reaction (Figure S7). On the contrary, for the compound (**1)**, up to 98% of Zn(Cy)_2_ is consumed in few minutes, and the simultaneous release of cyclohexane in the medium (peak at 1.45 ppm) evidences the hydrolysis of the zinc precursor. The interaction of Zn(Cy)_2_ with WO_3_·2H_2_O is schematized in Figure 8.

**Figure 9:**
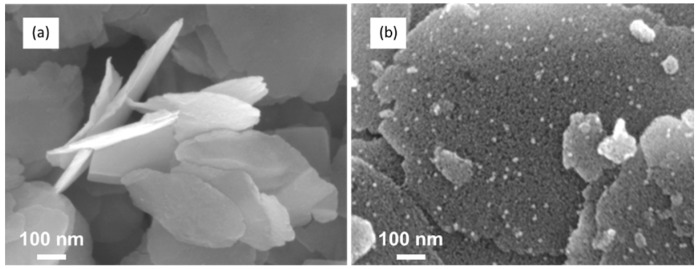
SEM images of (**1**) (**a**) pristine powder and (**b**) after mixing with 0.5 equiv. of Zn(Cy)_2_.

According to this result, we have concentrated our study on the reaction of the zinc precursor with (**1**) only. In order to master the amount of ZnO relative to the WO_3_ support, various Zn(Cy)_2_ contents (0.1, 0.25, 0.5 and 1 molar equivalent/W atom) were used in the reaction with (**1**). Figure 9 presents the SEM images of the NLs of (**1**) before and after being exposed, for 2 h, to a toluene solution containing 0.5 equiv. of Zn(Cy)_2_.

**Figure 10:**
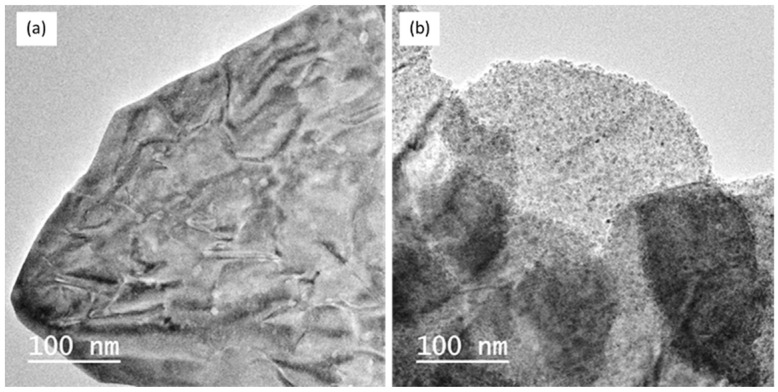
TEM images of (**1**) (**a**) pristine powder, and (**b**) after contact with 0.25 equiv. of Zn(Cy)_2_.

The growth of ZnO NPs on the NLs is clearly evidenced on the SEM images (Figure 9b,) where additional nanostructures appear in plane and on the edges of the WO_3_ supports. Similar features are revealed by TEM images (Figure 10a,b). The shape of the leaves is maintained during their decoration with ZnO NPs. The ZnO NPs size ranges from 4 to 8 nm; they are homogeneously distributed on the whole surface of the NLs. Interestingly, there is no free ZnO NPs present aside on the microscopy grid, which gives evidence of the exclusive growth of the nanocrystal over the WO_3_ supports. In addition, the powder was submitted to ten minutes ultrasonic treatment before the drop deposition on the microscopy grid. Even after this process, no difference is observed on the dispersion of ZnO on the NLs, and there are still no free ZnO NPs on the TEM grid. This suggests a robust interfacial contact between the ZnO NPs and the WO_3_ support.

**Figure 11:**
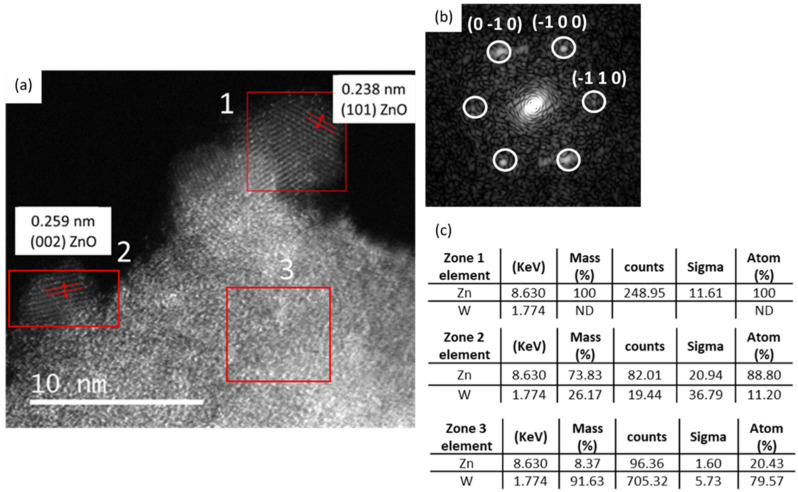
**(a**) HRTEM images of (**1**) exposed to 0.25 Zn(Cy)_2_ (**b**) FT image of the particle located in the zone 1 (indicated by red square in (**a**) showing Würtzite crystal plans, (**c**) EDS analysis of the zones 1, 2 and 3 delimitated by the red lines in the image.

HRTEM images of (**1**) modified by 0.25 equiv. of Zn(Cy)_2_ confirm the presence of the homogeneously dispersed nanostructures on the surface of WO_3_ leaves (Figure 11). The observation of the grains located at the extreme border of the WO_3_ supports clearly reveals their crystalline nature despite their low mean size (5 ± 2 nm) (Figure 11a). The FT study of such a ZnO nanoparticle (zone1) confirms the presence of crystalline planes corresponding to the Würtzite structure of ZnO (Figure 11b).

In addition to the previous analysis, EDX analyses performed on these nanosized structures reveal the exclusive presence of Zn oxide material (Figure 11c, zone 1). When the analyzed ZnO particles are too close to the WO_3_ substrate (zone 2) or located above the WO_3_ support (zone 3), the EDX analysis reveals the simultaneous presence of elements of both ZnO and the underlying WO_3_ support. This, therefore, confirms the growth of ZnO NPs at the surface of WO_3_ NLs.

**Figure 12:**
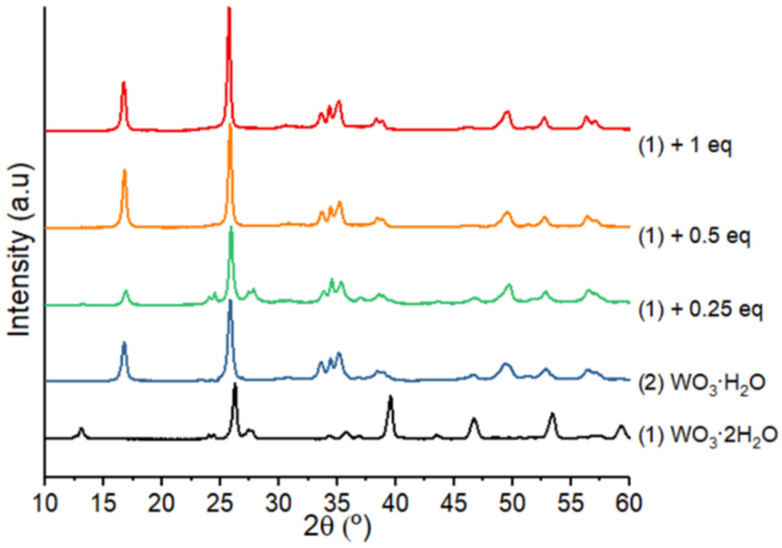
XRD diagrams of compounds (**1**) and (**2**), and (**1**) after mixing with 0.25, 0.5 and 1 equiv. of Zn(Cy)_2_.

XRD analyses performed on nanocomposites prepared with 0.25, 0.5 and 1 equiv. of. Zn(Cy)_2_ with (**1**) are presented in Figure 12. Remarkably, they all evidence the spontaneous phase transformation of (**1**) to (**2**) according to the disappearance of the 2Θ = 13.2° peak characteristic of (**1**). This result confirms that only one type of structural water molecule is used for the reaction with Zn(Cy)_2_ to produce ZnO. The most labile water molecule (interlamellar water molecules) are certainly involved in the reaction with Zn(Cy)_2_. The structural water molecule remaining within (**2**), corresponding to the type 2 (molecule coordinated into [WO_5_] octahedra), is therefore, not chemically accessible for the zinc precursor in the solution.

**Figure 13:**
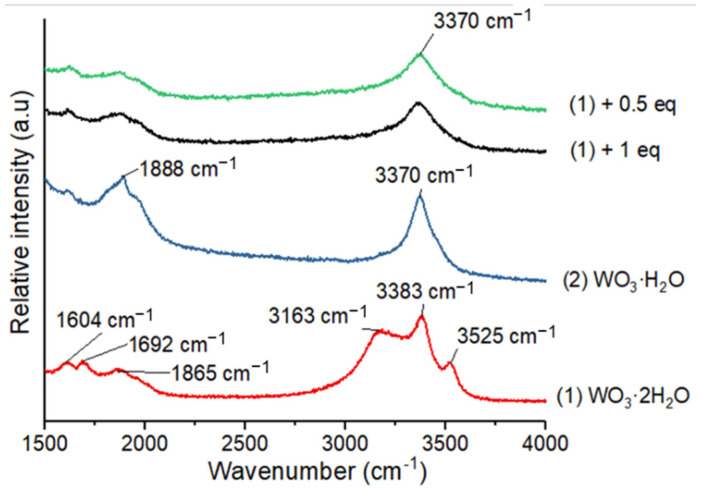
Raman spectra of (**1**) and (**2**) and (**1**) after mixing with 0.5 and 1 equiv. of ZnCy_2_.

However, no peak corresponding to a ZnO crystal structure is evidenced by XRD. The ZnO nanocrystals formed over the WO_3_ leaves are too small to enable any powder diffraction information. The structural evolution from (**1**) to (**2**) due to Zn(Cy)_2_ addition on (**1**) at room temperature is also confirmed by Raman, TGA and ^1^H MAS NMR analyses. The Raman analysis of compound (**1**) presents three bands at 3163, 3383 and 3525 cm^−1^ corresponding to the ν(H_2_O) vibrations of the two different structural water molecules (Figure 13).

After exposure to Zn(Cy)_2_, only one band at around 3370 cm^−1^ is evidenced in this region, similarly to the spectrum of the pure compound (**2**). As for XRD results, no additional bands corresponding to ZnO structure are evidenced by Raman analysis.

**Figure 14:**
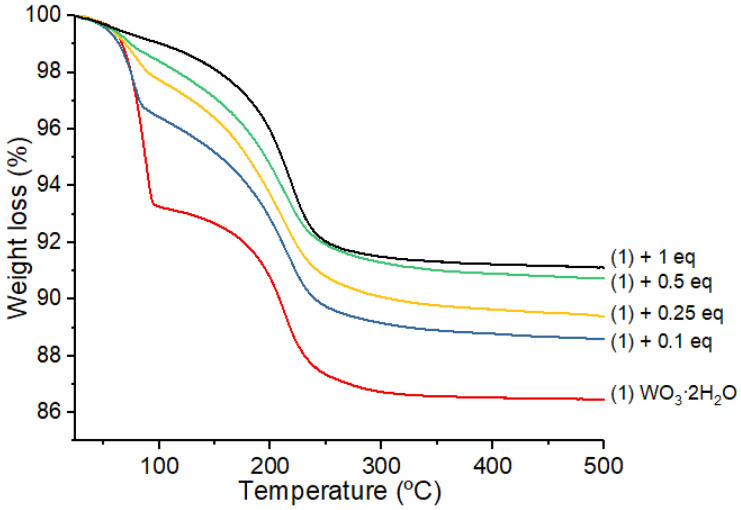
TGA analysis of (**1**) after exposition to 0.1, 0.25, 0.5 and 1 equivalent of Zn(Cy)_2_. The bottom curve corresponds to the TGA of pure WO_3_·2H_2_O (**1**) for reference.

TGA analyses were performed on various amounts of Zn(Cy)_2_ added to (**1**) in order to measure the quantity of water molecules consumed in the hydrolysis reaction with Zn(Cy)_2_ (Figure 14).

It clearly appears that only the most labile water molecules (those that leave at low temperature) are used during the reaction with Zn(Cy)_2_. The second water weight loss, beginning above 120 °C, remains quasi unaffected (around −6.6 to −7.9% weight loss) regardless of the amount of zinc precursor used (Table 1). This result confirms that the two types of water molecules of (**1**) are not equivalent in terms of reactivity or availability for a chemical reaction with Zn(Cy)_2_. The theoretical weight loss for one labile water molecule corresponds to 6.7% of the total mass of (**1**). A very close value (7.4%) is experimentally obtained by the TGA analysis of (**1**) (Table 1). When 0.1 equivalent of Zn(Cy)_2_ are reacted with (**1**), the weight loss drops down to 3.5%, i.e., up to 52% of the labile water molecules are consumed. With 0.5 and 1 equivalent of Zn(Cy)_2_, the weight losses are very similar and, respectively, drop to 1.54% and 1.20%, i.e., up to 78 and 82% of the labile water molecule were consumed by the zinc precursor. Therefore, with the 0.5 molar equivalent of Zn, close to 80% of the available type 1 water molecules are consumed, and there is only a little increase of the ZnO formation when doubling the amount zinc precursor (1 equiv.). In this reaction the limiting reacting agent is the quantity of available water molecules for the zinc precursor hydrolysis. When 0.1 molar equivalent of Zn(Cy)_2_ is employed, a large excess of labile water molecules (8 equiv.) in (**1**) is available for the reaction. Therefore, all the zinc precursor is consumed and a large amount of the water molecules remain on the support. With 0.5 equiv. of the zinc precursor, the quantity of excess water molecules is close to 1.7 equiv. Interestingly, this corresponds to the necessary amount of water experimentally determined for the full consumption of the zinc precursor when it is hydrolyzed in solution [17]. We can, therefore, hypothesize that in these conditions, almost all the zinc precursor is hydrolyzed and the maximum quantity of ZnO is grafted on the WO_3_ support. However, when 1 equiv. of Zn(Cy)_2_ is used, the quantity of water is at an unfavorable ratio (0.8 equiv.) to allow the full hydrolysis of zinc precursor. Therefore, the maximum of grafted ZnO corresponds to an amount close to 0.5 equiv. Adding extra zinc precursor will only generate unreacted product that will be eliminated by the washing sequence with toluene at the end of the process.

This is confirmed by the elementary analyses of Zn, W and O obtained by the electron probe microanalyzer presented in Table 1. The use of 0.5 and 1 equiv. of Zn(Cy)_2_ both lead to approximately 11 to 12% weight of Zn within the nanocomposite, which precisely corresponds to the presence of 0.5 mole of ZnO over WO_3_·H_2_O NLs. The maximum amount of grafted ZnO on WO_3_ is, therefore, limited by the necessary use of 2 equivalent of the available type 1 water molecules relative to the zinc precursor.

**Figure 15:**
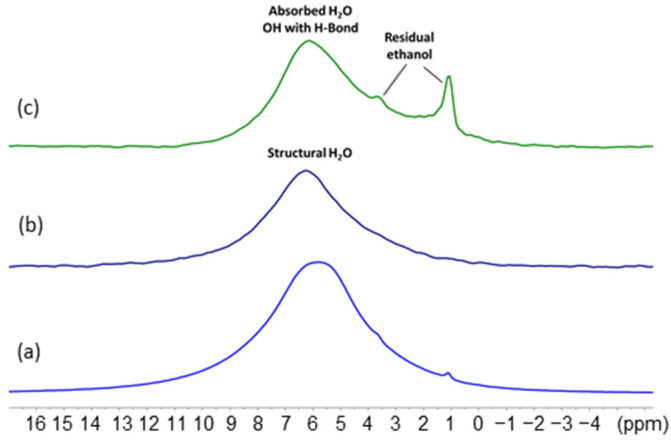
NMR spectra of (**1**) after exposition to 0.25 equiv. of Zn(Cy)_2_. (**a**) ^1^H MAS spectrum, (**b**) DQ MAS spectrum evidencing the signal of the remaining structural water molecule, (**c**) DF-SE MAS spectrum showing the presence of mobile OH groups in the compound. Peaks at 3.6 ppm and 1.1 ppm can be due to ethanol residue after washing and centrifugation of the sample.

MAS NMR analyses of (**1**) after reaction with 0.25 equiv. of Zn(Cy)_2_ confirm the above described results. The ^1^H spectrum (Figure 15a) reveals a single and broad peak centered at 5.8 ppm, similar to the one obtained for (**2**), which arises notably from the remaining structural water molecules. The 1D DQ MAS NMR signal at 6.3 ppm also confirms the presence of rigid H atoms pairs related to structural H_2_O molecules (Figure 15b). The complementary NMR experiment with the dipolar filter (DF-SE) that highlights the mobile or isolated H atoms in the structure, also gives a strong signal at 6.1 ppm (Figure 15c). This signal can be attributed to mobile physisorbed water molecules and/or to isolated OH groups engaged in a moderate H-Bond.

### 3.3. Transformation of ZnO@WO_3_ Nanocomposite into ZnWO_4_ @WO_3_

**Figure 16:**
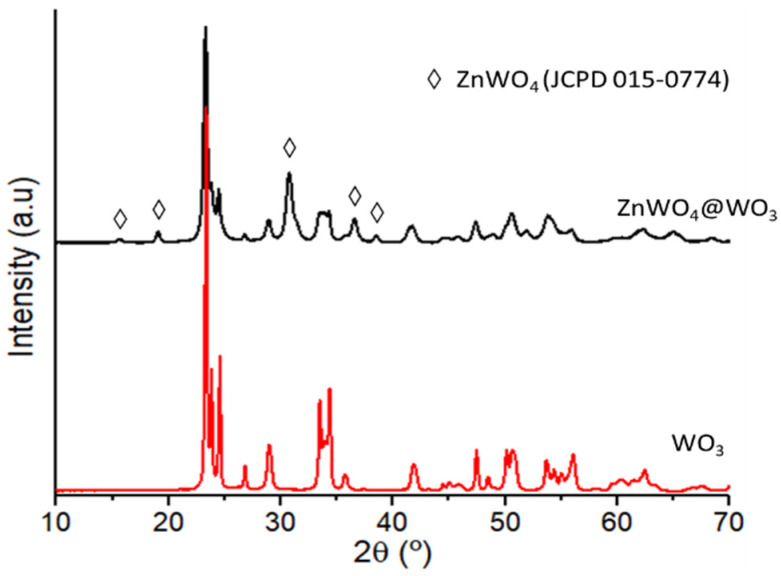
XRD analyses of the composite ZnO@WO_3_.H_2_O (0.5 equiv. Zn(Cy)_2_) annealed at 500 °C in air. The diffraction peaks correspond to the well crystallized WO_3_ monoclinic phase. The peaks corresponding to the additional ZnWO_4_ phase are marked with a diamond.

A thermal treatment of the nanocomposite material was performed up to 500 °C in air in order to fully remove the water molecules from the WO_3_ support and also to improve the crystallinity of the material. Indeed, crystallinity plays a significant role for improving the photocatalysis processes [37,38]. However, during the thermal annealing, the ZnO nanocrystals react with the WO_3_ support to yield a new mixed oxide ZnWO_4_ phase (JCPD 00-015-0774, monoclinic). The XRD analysis of the composite prepared with 0.5 equiv. of Zn(Cy)_2_ with (**1**) and thermal treated at 500 °C is presented in Figure 16. It confirms the simultaneous presence of well crystallized monoclinic WO_3_ support that remains oriented along the basal (002) planes, in addition to the additional ZnWO_4_ phase.

**Figure 17:**
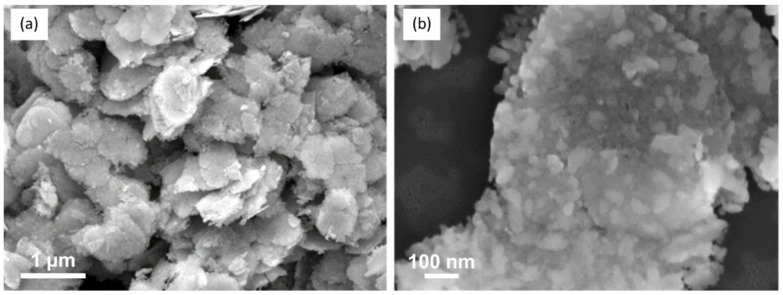
SEM images of ZnWO_4_@WO_3_ composite: (**a**) low magnification image (×20,000) showing the NL grains, (**b**) high magnification image (×100,000) showing the ZnWO_4_ grains on the WO_3_ NLs.

This evolution is confirmed by Raman analysis of the compound prepared with 1 equiv. Zn(Cy)_2_ and thermally treated at 500 °C (Figure S8). The analysis evidences the simultaneous presence of bands at 791, 693 and 257 cm^−1^ relative to WO_3_ and several bands at 897, 402, 320 and 185 cm^−1^ corresponding to the ZnWO_4_ wolframite phase [39]. Note that the redshift of bands compared to the positions reported in the literature at room temperature are due to the effect of temperature. During this thermal treatment the WO_3_ grain morphology has evolved towards more aggregated structures, as evidenced by SEM images (Figure 17). However, they still present leaf-shape structures. In addition, the nanosized ZnO features initially present at their surface have disappeared to the benefit of much larger ZnWO_4_ grains (*ca.* 40 nm) homogeneously covering the WO_3_ leaves.

This new nanocomposite is of great interest for catalytic degradation of pollutants [11,40,41] and the photocatalytic tests for NO_2_ degradation are presented hereafter.

### 3.4. Photocatalytic Properties of the WO_3_-Based Nanocomposites for NO_2_ Degradation

Combining WO_3_ and ZnWO_4_ with matching energy level configuration is known to form a type II (staggered) heterojunction that is useful for increasing the lifetime and number of photogenerated holes compared to pristine WO_3_ [40,41]. In addition, Au NP deposition is often used to improve the overall photocatalytic performances of photocatalytic metal oxides MOx [42,43]. The formation of Au-MOx interfaces allows a faster photogenerated charge carrier transfer and, therefore, improves the spatial separation of electron/holes pairs in the material. As a consequence, a good interface reduces the recombination rate of photogenerated charges and eventually enhances chemical reactions at the nanocomposite surface. In our case Au NPs (1% at.) were deposited on WO_3_ and ZnWO_4_@WO_3_ powders by a simple photodecomposition of the HAuCl_4_ precursor in solution (see Material and Methods section) [44]. The Au decoration of WO_3_ and ZnWO_4_@WO_3_ nanocomposites is evidenced by TEM and SEM (Figures S9 and S10). The Au NPs present a mean diameter of 22 ± 14 nm and 17 ± 10 nm, respectively (Figure S11), and are evenly distributed over the oxide surface.

**Figure 18:**
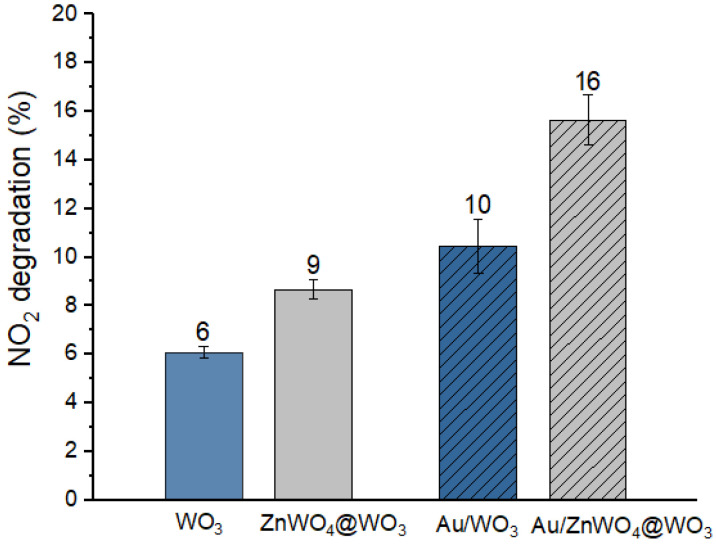
Photocatalytic NO_2_ degradation obtained with WO_3_, ZnWO_4_@WO_3_, Au/WO_3_ and Au/ZnWO_4_@WO_3_ nanomaterials deposited on glass substrates under UV-A irradiation.

The photocatalytic performances of the nanocomposites were investigated for the degradation of NO_2_ under UV radiation by a UV-A (Figure S1, λ = 365 nm, 1 W/m^2^) light source at room temperature. This reaction has rarely been studied under low NO_2_ concentration of and low UV irradiation [45]. In addition, according to Gandolfo et al. [46], a UV-A irradiation below 5 W/m^2^ is enough for enabling NO_2_ photocatalytic degradation whilst reducing unwanted by-product generation such as NO and nitrous acid (HONO). The photocatalytic abatement of NO_2_ under UV-A with the different nanocomposites of this study, WO_3_, ZnWO_4_@WO_3_, Au/WO_3_ and Au/ZnWO_4_@WO_3_, are presented in Figure 18.

Note that a slight loss of the NO_2_ removal efficiency (1–2%) is measured when several tests are run consecutively. This decrease may be associated to the possible NO_3_^−^ adsorption at the photocatalyst surface, resulting in the blockage of some of the catalytically active sites. Longer tests (>2 h) were not included in the scope of this work, but they will be carried out in a forthcoming study to assess the long duration NO_2_ abatement properties of the nanocomposites. It is noteworthy that the samples recover their initial NO_2_ abatement performance after storage in the dark at ambient air for 24 h.

The degradation of NO_2_ is clearly improved by (i) Au NP deposition, whatever the oxide surface; and (ii) the presence of ZnWO_4_ nanostructures over WO_3_. The presence of ZnWO_4_ alone on WO_3_ already leads to a 50% improvement in the NO_2_ abatement compared to pure WO_3_. The level of improvement is close to the one achieved by Au decoration of WO_3_ NLs (+66%). The best photocatalyst of this study is the most complex nanocomposite Au/ZnWO_4_@WO_3_, which allows an improvement of 166% of the abatement of NO_2_ compared to pure WO_3_. Both Au and ZnWO_4_ are known to present a good chemical affinity to NO_2_, which allows improvement of the absorption of the pollutant and the chemical transformation at the material surface [47,48]. In addition to these better adsorption properties, the interaction of WO_3_ with Au and ZnWO_4_ also induces important electronic property changes in the material. Indeed, WO_3_ is known for its very fast recombination of charge carrier leading to a lower photocatalytic degradation efficiency compared to other metal oxides such as TiO_2_ or ZnO [49]. Conversely, ZnWO_4_ presents a high ionic conductivity and long lifetime of photogenerated carriers under UV irradiation [12,49,50]. In addition to this effect, Au NP grafted on the metal oxide surface help to improve the separation and transfer of photogenerated charges from the nanocomposites. Indeed, the hydroxyl radical (OH^•^) are the main active species for the degradation of NO_2_ according to the following reaction:

(2) $${\mathrm{NO}}_{2}+{\mathrm{HO}}^{•}\to {\mathrm{NO}}_{3}^{-}+H^{+}$$

**Figure 19:**
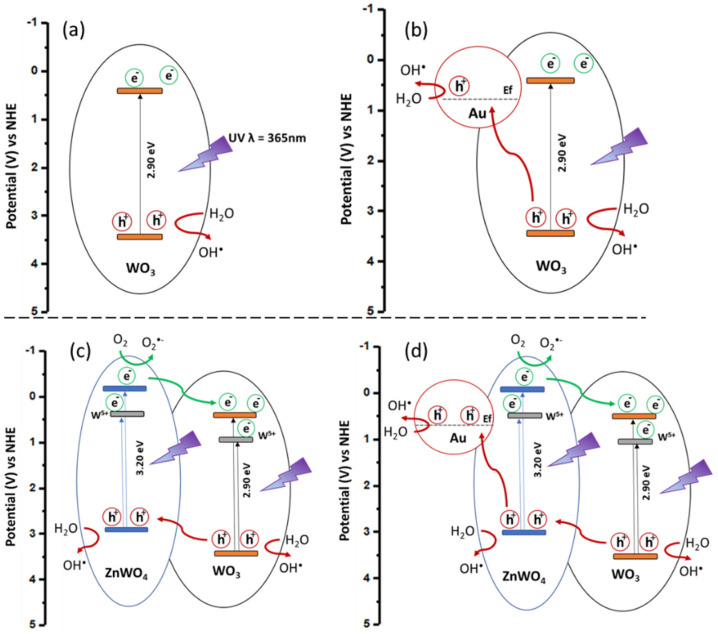
Energy diagrams, charge separation and hydroxyl radicals (OH^•^) formation under UV-A irradiation for (**a**) WO_3_, (**b**) ZnWO_4_@WO_3_, (**c**) Au/WO_3_ and (**d**) Au/ZnWO_4_@WO_3_ nanocomposites.

The mechanisms for the production of OH^•^ radicals in WO_3_-based composites for NO_2_ degradation are summarized in Figure 19. Figure 19a shows the photocatalytic OH^•^ production of pristine WO_3_ material [51]. The improvement of the photocatalytic efficiency of this n type metal oxide is achieved by (i) Au NP decoration (Figure 19b) and (ii) by the creation of an interface with ZnWO_4_ (Figure 19c). Indeed, both these materials act as fast h^+^ trapping centers [51,52] and provide more active sites to produce a higher number of OH^•^ species. The heterojunction between ZnWO_4_ and WO_3_ also leads to a better transfer and separation of the photogenerated electron–hole pairs. In addition, the ZnWO_4_-WO_3_ interface induces more oxygen vacancies and W^5+^ species that behave as a reservoir for photogenerated electrons [12,53,54,55]. These species participate in the decrease in the charge recombination reactions and, finally, improve the overall photocatalytic performances of the nanocomposite. Interestingly, ZnWO_4_, which presents a higher band gap (3.2 eV) compared to WO_3_ (2.9 eV), also displays a more negative conduction band potential (≈−0.8 eV) [56] able to produce O_2_^−^ radicals [56,57,58] (Figure 19c). These photogenerated species also act as electron reservoirs, thus, limiting the recombination rate and eventually improving the OH^•^ surface density. The addition of both (i) and (ii) effects in the Au/ZnWO_4_@WO_3_ complex hetero-nanomaterial (Figure 19d) is the best combination to produce a high quantity of hydroxyl radical and, therefore, an optimized NO_2_ removal under UV illumination.

## 4. Conclusions

In this work we have proposed a new chemical strategy leading to the very localized growth of ZnO nanostructures over WO_3_·2H_2_O NLs. We have highlighted the role of specific water molecules in the WO_3_·2H_2_O crystalline geometry that are labile enough to react with the organometallic zinc precursor and allow its localized hydrolysis. After hydrolysis, the ZnO nanostructures are supported on the mono hydrate WO_3_·H_2_O compound. A thermal annealing at 500 °C in air was applied on the nanocomposite in order to remove the remaining structural water and generate well-crystallized WO_3_ photocatalytic oxide. During this treatment, ZnO diffuses within the WO_3_ supporting oxide to generate the nanocomposite ZnWO_4_@WO_3_. The core of the NLs remains composed of WO_3_ nanocrystals, as shown by XRD and Raman analyses. This nanocomposite was further decorated with Au NP for the assessment of photocatalytic abatement of 400 ppb NO_2_ in air under a UV-A source. In these conditions, the complex hetero-nanomaterial Au/ZnWO_4_@WO_3_ shows a 166% increase in NO_2_ degradation performance compared to pristine WO_3_ NLs powder. This result suggests the presence of intimate ZnWO_4_/WO_3_ interfaces (type II heterojunction), allowing a better efficiency in charge carrier separation and eventually leading to a higher photocatalytic activity thanks to OH^•^ radicals. This synthetic route for building complex hetero-nanomaterial can be extended to many other oxide combinations dedicated to a wide range of applications, from catalysis to gas sensing devices. The composition of the heterostructures will depend on the careful choice of the organometallic precursors and the chemical composition of the supporting oxide that should exhibit chemically available water molecules at its surface.

## Figures and Tables

**Table 1:** TGA and elementary analyses of (**1**) after reaction with increasing amounts of Zn(Cy)_2_.

		TGA		Microanalysis		
Zn(Cy)_2_ Amount	Molar Ratio H_2_O/Zn	Low T° H_2_O Weight Loss (%)	High T° H_2_O Weight Loss (%)	Zn (% wt.)	W (% wt.)	O (% wt.)
0	-	7.4	8	-	76.6	19.9
0.1	8	3.5	7.8	-	-	-
0.25	3.4	2.2	8	6.8	65	23
0.5	1.7	1.54	7.9	11.2	61	24.2
1	0.8	1.2	8.1	12.2	60.5	23

## Data Availability

Data are contained within the article.

## Supplementary Materials

The following supporting information can be downloaded at: https://www.mdpi.com/article/10.3390/nano12244360/s1, Figure S1. Irradiance spectrum of the NARVA Blacklight Blue fluorescent tube used for NO_2_ degradation experiments; Figure S2. (a) Arrangement scheme of [WO_5_·H_2_O] octahedrons in the WO_3_·2H_2_O compound; (b) View of the arrangement along *c* axe showing the two types of H_2_O (i) and H_2_O (c) molecules for intermediate and coordinated H_2_O respectively; Figure S3. Raman analysis of (**1**) during in situ heating up to 120 °C; Figure S4. ^1^H RFDS MAS NMR spectra of (**1**), (**2**) and (**3**); Figure S5. 1H DQ MAS NMR spectra of (**1**), (**2**) and (**3**); Figure S6. TEM images of WO_3_·xH_2_O NL after reaction with Zn(Cy)_2_. (a) x = 2: A high density of nanosized structures appear at the surface of WO_3_; (b) x = 1: no modification of the WO_3_ surface; (c) x = 0; similar to x = 1: no modification of the WO_3_ surface; Figure S7. ^1^H NMR study of Zn(Cy)_2_ (I and II) and in contact with WO_3_·xH_2_O NL (1) x = 2 (I, II and III); (2) x = 1 and (3) x = 0. The pic of toluene-d^8^ (solvent) and the cyclohexane generation are indexed. No evolution of Zn(Cy)_2_ appears when placed in contact with WO_3_·H_2_O or WO_3_; Figure S8. Raman analysis of ZnWO_4_@WO_3_ nanocomposite annealed at 500 °C; Figure S9. Size distribution diagrams of Au NPs measured on TEM image (*n* = 150) of (a) Au/WO_3_ and (b) Au/ZnWO_4_/WO_3_; Figure S10. TEM image of (a) Au/ZnWO_4_@WO_3_ composite and (b) Au/WO_3_; Figure S11. SEM-BSE images of (a) and (b) Au/ZnWO_4_@WO_3_ composite and (c) Au/WO_3_. The bright points indicated by arrows correspond to Au nanoparticles.
