# Supplementary material for: Nano-Structuration of WO3 Nanoleaves by Localized Hydrolysis of an Organometallic Zn Precursor: Application to Photocatalytic NO2 Abatement
Source: Nanomaterials (Basel). 2022 Dec 7;12(24):4360. doi: 10.3390/nano12244360 (PMC9786007; doi:10.3390/nano12244360)
Supplement: Supplementary file 1 [file nanomaterials-12-04360-s001.zip › nanomaterials-2072819-supplementary.pdf]

Nano-Structuration of WO_3 Nanoleaves by Localized Hydrolysis of an Organometallic Zn Precursor: Application to Photocatalytic NO_2 Abatement

Kevin Castello Lux [1,2], Katia Fajerwerg [2,3], Julie Hot [1], Erick Ringot [1,4], Alexandra Bertron [1], Vincent Collière [2,3], Myrtil L. Kahn [2], Stéphane Loridant [5], Yannick Coppel [2] and Pierre Fau [6,3,*]

[1] LMDC, INSA/UPS Génie Civil, 135 Avenue de Rangueil, CEDEX 4, 31077 Toulouse, France
[2] LCC-CNRS, UPR8241, 205 Route de Narbonne, CEDEX 4, 31077 Toulouse, France
[3] Université de Toulouse, UT3 Paul Sabatier, 118 Route de Narbonne, CEDEX 04, 31062 Toulouse, France
[4] LRVision SAS, 13 Rue du Développement, 31320 Castanet-Tolosan, France
[5] Univ. Lyon, Université Claude Bernard-Lyon 1, CNRS, IRCELYON-UMR 5256, 2 av. A. Einstein, 69626 Villeurbanne, France
[6] LPCNO-INSA, UMR5215, 135 Avenue de Rangueil, CEDEX 4, 31077 Toulouse, France
* Correspondence: pfau@insa-toulouse.fr

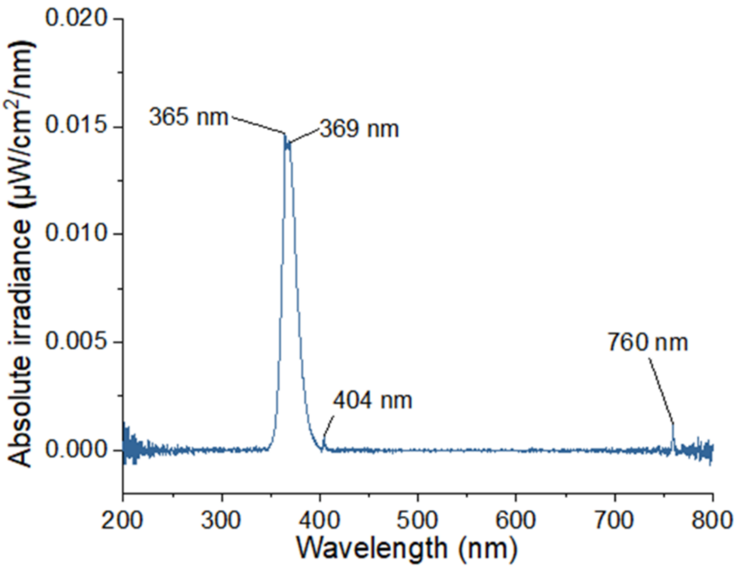

Figure S1. Irradiance spectrum of the NARVA Blacklight Blue fluorescent tube used for NO_2 degradation experiments.

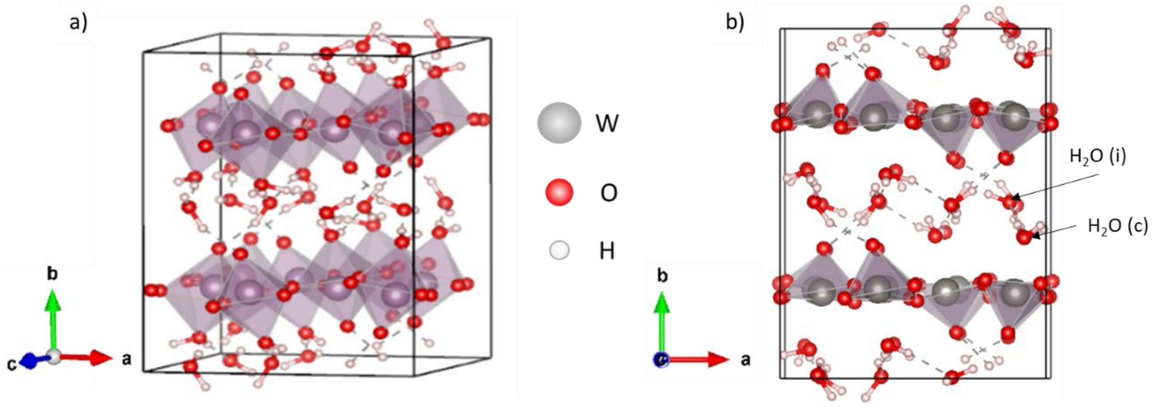

Figure S2. a) Arrangement scheme of [$WO_5.H_2O$] octahedrons in the $WO_3.2H_2O$ compound. b) View of the arrangement along *c* axe showing the two types of H_2O (i) and H_2O (c) molecules for intermediate and coordinated H_2O respectively.

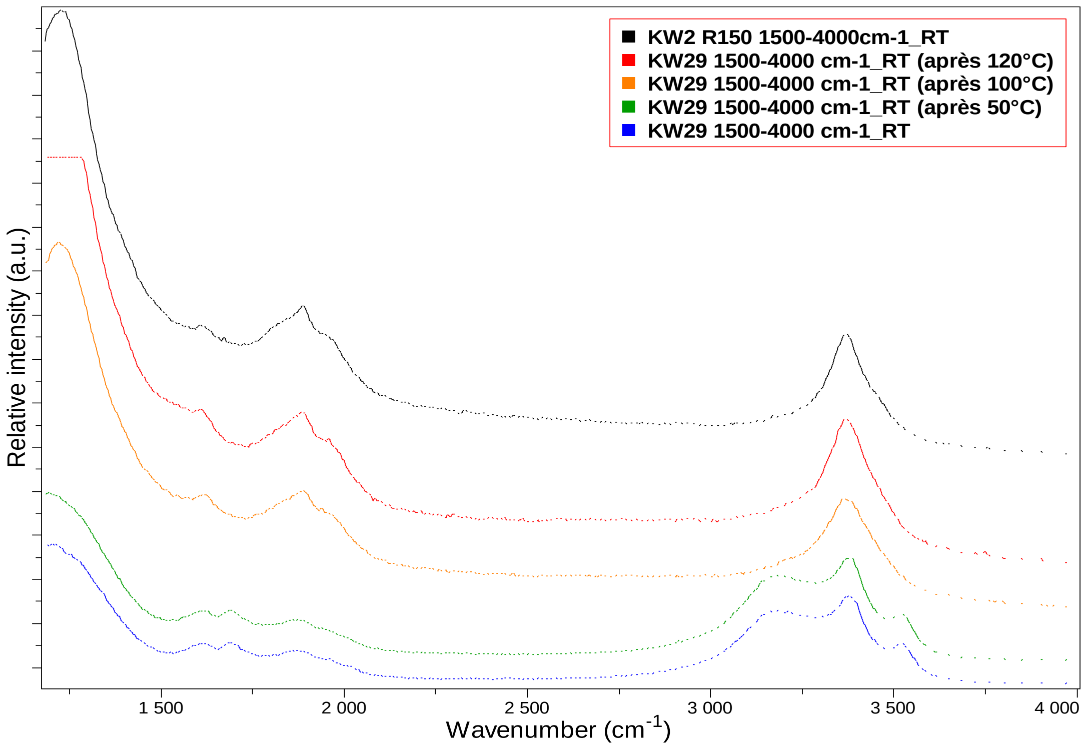

Figure S3. Raman analysis of (**1**) during in situ heating up to 120 °C.

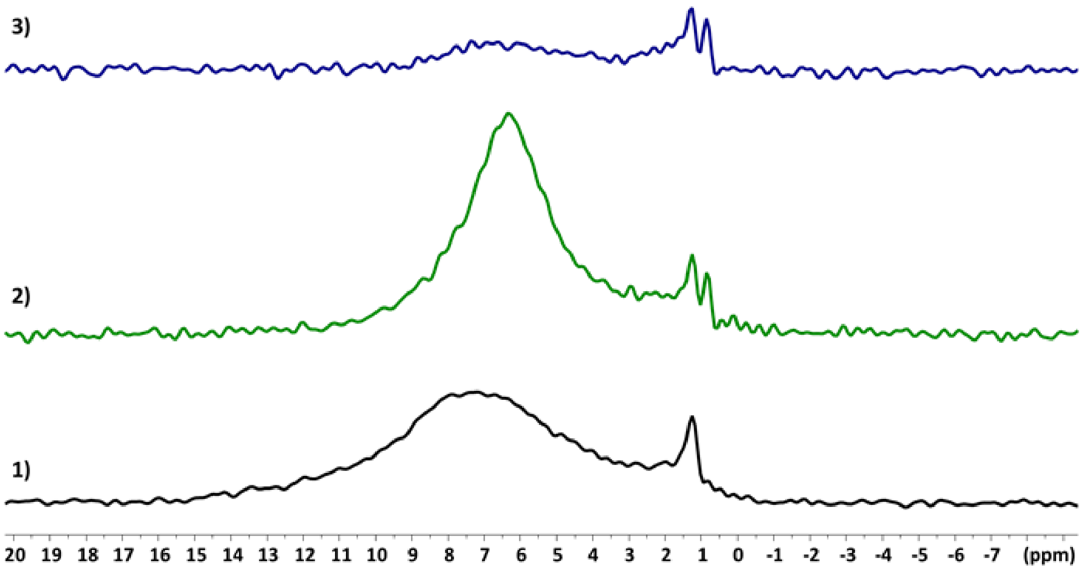

Figure S4. ^{1}H RFDS MAS NMR spectra of (**1**), (**2**) and (**3**)

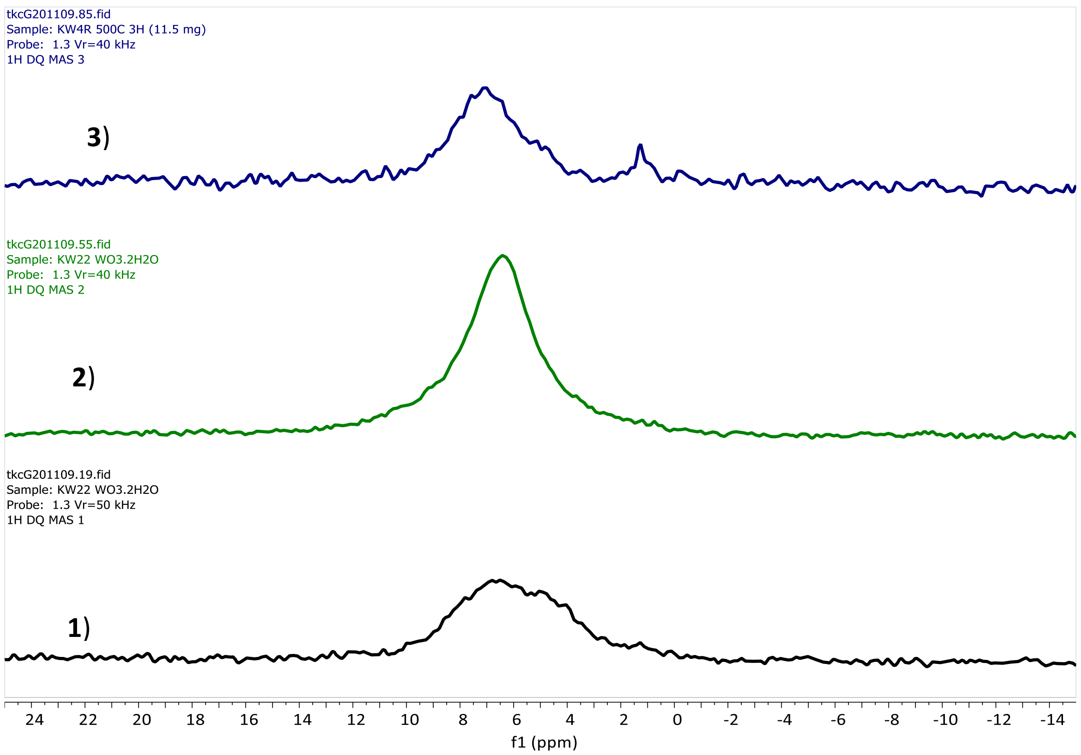

Figure S5. 1H DQ MAS NMR spectra of (**1**), (**2**) and (**3**)

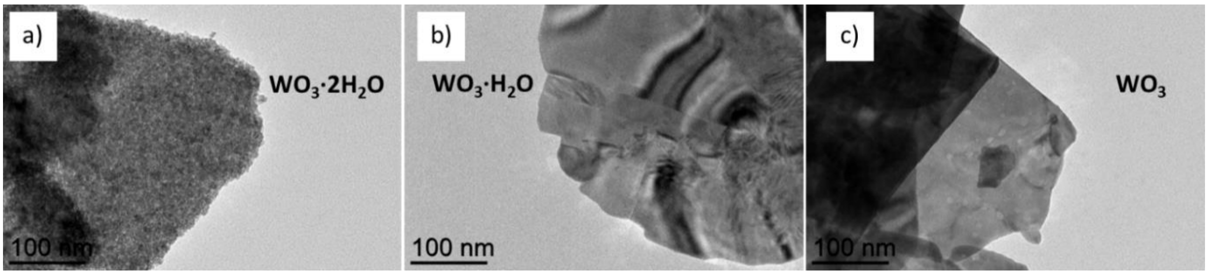

Figure S6. TEM images of $WO_3 \cdot xH_2O$ NLs after reaction with $Zn(Cy)_2$. a) x=2: a high density of nanosized structures appear at the surface of WO_3; b) x=1 : no modification of the WO_3 surface; c) x=0: no modification of the WO_3 surface.

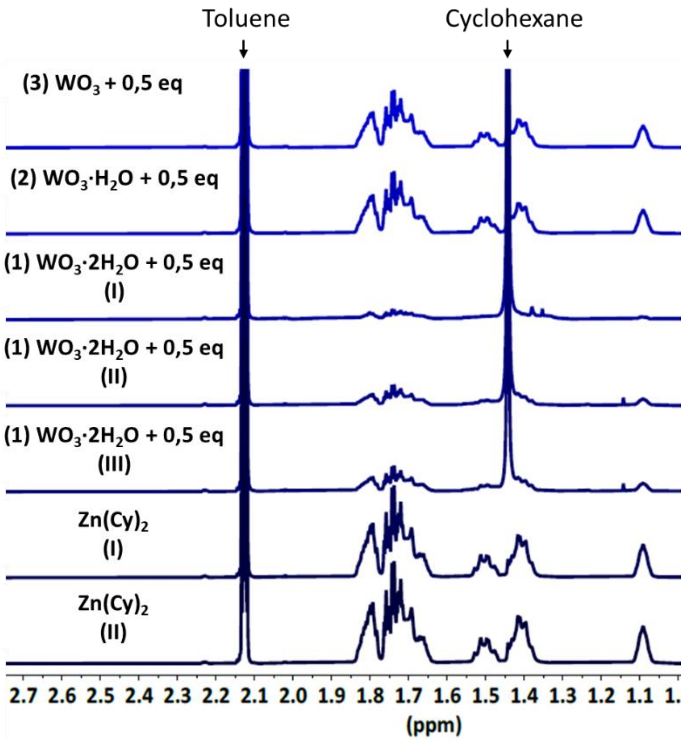

Figure S7. ^{1}H NMR study of $Zn(Cy)_2$ (I and II) and in contact with WO_3.x H_2O NL 1) x=2 (I, II and III); 2) x=1 and 3) x=0. The pic of toluene-d^8 (solvent) and the cyclohexane generation are indexed. No evolution of $Zn(Cy)_2$ appear when it is mixed with $WO_3.H_2O$ or WO_3.

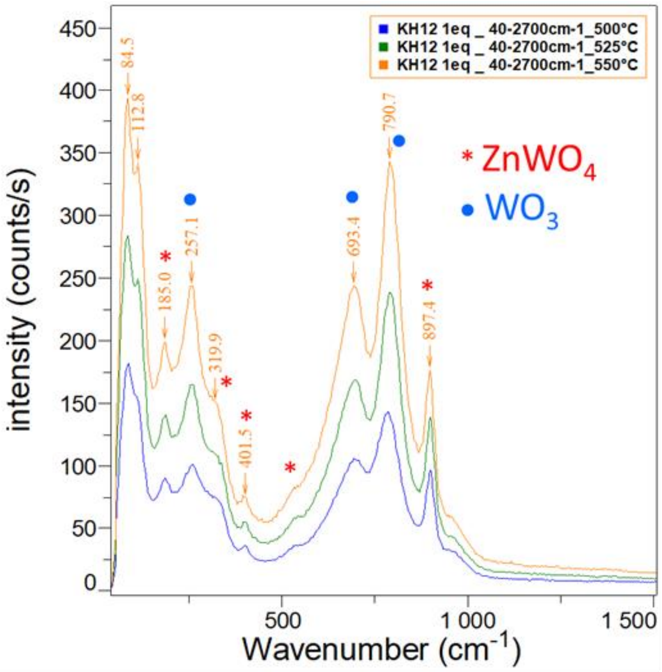

Figure S8. Raman spectra of $ZnWO_4@WO_3$ nanocomposite annealed at 500 °C

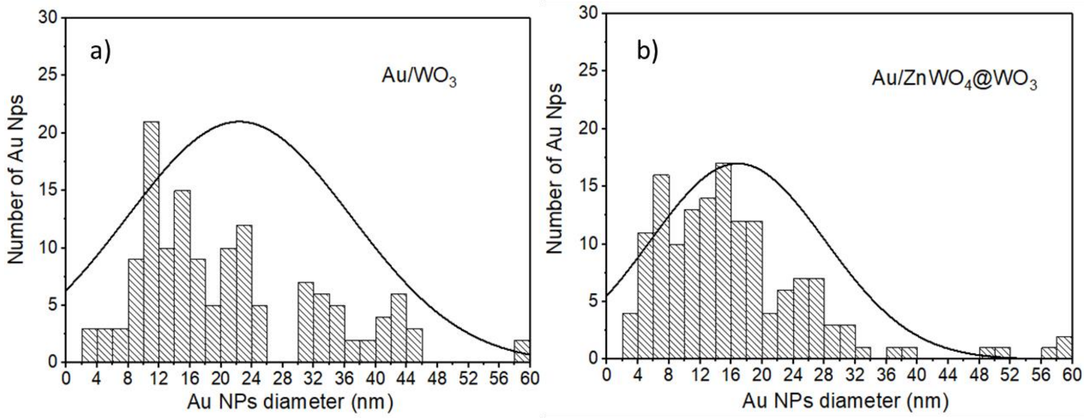

Figure S9. Size distribution diagrams of Au NPs measured on TEM image (n = 150) for a) Au/WO_3 and b) Au/$ZnWO_4$/WO_3

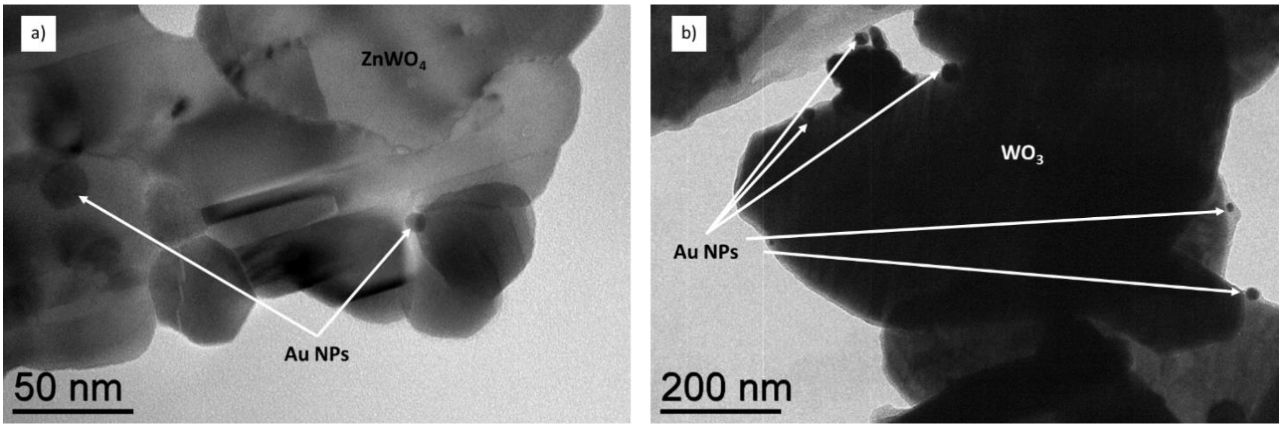

Figure S10. TEM image of a) Au/$ZnWO_4$@WO_3 nanocomposite and b) Au/WO_3

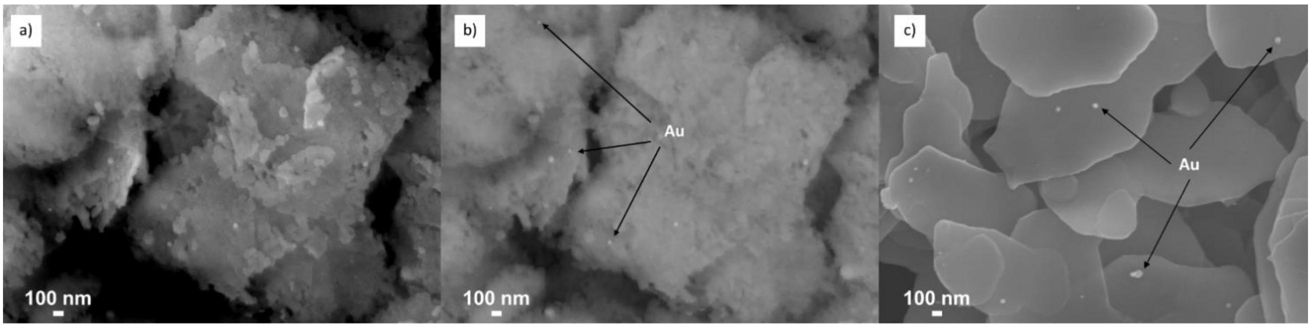

Figure S11. SEM-BSE images of a) and b) Au/$ZnWO_4$@WO_3 composite and c) Au/WO_3. The bright points indicated by arrows correspond to Au nanoparticles.
